# RLIP76, a Glutathione-Conjugate Transporter, Plays a Major Role in the Pathogenesis of Metabolic Syndrome

**DOI:** 10.1371/journal.pone.0024688

**Published:** 2011-09-13

**Authors:** Jyotsana Singhal, Lokesh Nagaprashantha, Rit Vatsyayan, Sanjay Awasthi, Sharad S. Singhal

**Affiliations:** 1 Department of Diabetes and Metabolic Diseases Research, Beckman Research Institute, City of Hope, National Medical Center, Duarte, California, United States of America; 2 Department of Molecular Biology and Immunology, University of North Texas Health Science Center, Fort Worth, Texas, United States of America; Roswell Park Cancer Institute, United States of America

## Abstract

**Purpose:**

Characteristic hypoglycemia, hypotriglyceridemia, hypocholesterolemia, lower body mass, and fat as well as pronounced insulin-sensitivity of RLIP76^−/−^ mice suggested to us the possibility that elevation of RLIP76 in response to stress could itself elicit metabolic syndrome (MSy). Indeed, if it were required for MSy, drugs used to treat MSy should have no effect on RLIP76^−/−^ mice.

**Research Design and Methods:**

Blood glucose (BG) and lipid measurements were performed in RLIP76^+/+^ and RLIP76^−/−^ mice, using Ascensia Elite Glucometer® for glucose and ID Labs kits for cholesterol and triglycerides assays. The ultimate effectors of gluconeogenesis are the three enzymes: PEPCK, F-1,6-BPase, and G6Pase, and their expression is regulated by PPARγ and AMPK. The activity of these enzymes was tested by protocols standardized by us. Expressions of RLIP76, PPARα, PPARγ, HMGCR, pJNK, pAkt, and AMPK were performed by Western-blot and tissue staining.

**Results:**

The concomitant activation of AMPK and PPARγ by inhibiting transport activity of RLIP76, despite inhibited activity of key glucocorticoid-regulated hepatic gluconeogenic enzymes like PEPCK, G6Pase and F-1,6-BP in RLIP76^−/−^ mice, is a salient finding of our studies. The decrease in RLIP76 protein expression by rosiglitazone and metformin is associated with an up-regulation of PPARγ and AMPK.

**Conclusions/Significance:**

All four drugs, rosiglitazone, metformin, gemfibrozil and atorvastatin failed to affect glucose and lipid metabolism in RLIP76^−/−^ mice. Studies confirmed a model in which RLIP76 plays a central role in the pathogenesis of MSy and RLIP76 loss causes profound and global alterations of MSy signaling functions. RLIP76 is a novel target for single-molecule therapeutics for metabolic syndrome.

## Introduction

Metabolic syndrome (MSy), a condition characterized by impaired inhibition of hepatic gluconeogenesis, insulin-resistance, hypercholesterolemia, hypertriglyceridemia, hyperglycemia, obesity, hypertension, and fatty-liver occur simultaneously, has reached epidemic proportions in the USA, and represents a very substantial burden on health care [1]–[5]. Also increasing in incidence is type II diabetes mellitus (T2D), associated with hyperglycemia, insulin-resistance, and hyperlipidemia [3], [6], [7]. MSy is frequently seen in survivors of HIV, cancer survivors treated previously with chemotherapy and/or radiation, and as a side effect of chronic toxicity of certain medications, such as antipsychotic agents. MSy and T2D are cardiovascular risk factors, and their treatment reduces cardiovascular risk, but requires combinations of hypoglycemic agents, statins, fenofibrate, and niacin that together pose a significant therapeutic challenge related to patient compliance, toxicities and drug-interactions [8]–[10].

There is a strong correlation between oxidative-stress and acquired insulin-resistance, and the pathological conditions that are associated with oxidative stress frequently result in hyperglycemia [11]–[13]. Glutathione (GSH) and GSH-linked metabolic pathways are a primary defense against oxidative stress [14], [15]. Oxidative stress is implicated as a contributor to pancreatic β-cell-apoptosis because of their susceptibility to the damaging effects of free radicals due to low levels of free radical quenching enzymes including catalase, glutathione-peroxidase (GPx) and superoxide dismutase [11], [14]–[16]. Oxidative stress generated by a short exposure of β-cells to H_2_O_2_ results in decrease in insulin mRNA level, and consequent suppression of insulin secretion [17], [18]. Moreover, exposure of β-cells to high glucose concentration induces generation of intracellular free radicals causing the inhibition of insulin release [19]. Prolonged exposure to oxidative stress affects transcription of glucose transporter GLUT4 through inhibition of binding of nuclear proteins to the insulin-responsive element in the GLUT4 promoter [20]. These and other mechanisms have been proposed as causes of insulin-resistance, but a unified model has not emerged that adequately explains the mechanisms through which clinically used drugs reverse insulin resistance, which is a characteristic of MSy and T2D.

Important and effective oral hypoglycemic agents that function through transcriptional regulation of gluconeogenic and lipid-metabolizing enzymes in the liver induce their effects either indirectly or directly by mechanisms that are mediated through peroxisome proliferator-activated receptors γ (PPARγ) and AMP-activated protein kinase (AMPK). Rosiglitazone (Avandia) and pioglitazone (Actos) are thiazolidinedione-class (TZD) oral hypoglycemic agents whose primary mechanism is through PPARγ mediated transcriptional regulation of carbohydrate and lipid metabolism [21]. Metformin (Glucophage) is a biguanide-class hypoglycemic agent that operates primarily through inhibition of AMPK (which regulates the rate of endocytosis), but is also linked with PPARγ through PGC1α (PPARγ-receptor co-activator) [22]. These medications generally function to increase the effectiveness of insulin-mediated postprandial inhibition of hepatic gluconeogenesis. Increasingly, the beneficial effects of these medications have been linked with their ability to act as antioxidants or to induce biological antioxidant defenses [21], [22].

During our studies of a mercapturic acid pathway transporter protein (RLIP76, ral-interacting protein) which is involved in protecting cells from oxidative and electrophilic stress, we discovered that RLIP76 homozygous knockout (RLIP76^−/−^) mice had blood glucose, triglyceride and cholesterol levels that were each approximately half of those found in wild-RLIP76 (RLIP76^+/+^) mice. Because these mice had decreased body fat as well as marked central and peripheral insulin-sensitivity, they appeared in essence the diametric opposite of MSy [1]. This observation is even more remarkable considering that the “anti-MSy” phenotype of these animals occurs despite the fact that lipid-peroxidation levels in the tissues of these mice are 2–7 fold higher than wild-type mice [23]–[26]. That is, conditions (i.e oxidative-stress) that lead to insulin-resistance in the presence of RLIP76 do not have this effect in its absence. These considerations led us to hypothesize that RLIP76 represents a required mechanism to translate the presence of markedly increased levels of oxidative-stress into insulin-resistance and hyperlipidemia. A corollary to this hypothesis is that medications commonly used to treat hyperglycemia and hypertriglyceridemia should fail to function in RLIP76^−/−^ mice if RLIP76 is indeed necessary for the clinical manifestations of oxidative-stress characteristic of MSy and T2D. In present studies, we describe the findings of studies with rosiglitazone, metformin, atorvastatin and gemfibrozil, showing that these drugs indeed entirely lack hypoglycemic or hypolipidemic effects in RLIP76^−/−^ mice. Results of present studies thus offer conclusive evidence for a critical role of RLIP76 as an effector protein directly involved in translating oxidative-stress into the clinical manifestations of MSy and T2D. Implications of our findings include the possibility of single RLIP76-targeted molecules that can have broad salutary affects in T2D as well as MSy.

## Materials and Methods

### Materials

Gemfibrozil and metformin were purchased from Sigma Chemical (St Louis, MO). Rosiglitazone and atorvastatin (lipitor) were purchased from Cayman Chemical (Ann Arbor, MI) and LC Laboratories (Woburn, MA), respectively. PPARα, PPARγ, pAMPK, pAkt, pJNK, and hydroxyl-3-methylglutaryl coenzyme A reductase (HMGCR) antibodies were purchased from Santa Cruz Biotechnology and Upstate Cell Signaling. Blood glucose was measured by Ascensia Elite Glucometer®. Blood cholesterol and triglycerides assay kits were procured from ID Labs (London, ON, Canada). Blood glucose, cholesterol and triglycerides measurement were also performed and validated in the laboratory of Dr. Kent R. Refsal, Michigan State University, Michigan. Avidin/biotin complex (ABC) detection/staining kit were purchased from Vector (Burlingame, CA).

### Animals

RLIP76^+/−^ heterozygous knockout mice were generated by Lexicon Genetics, The Woodlands, TX [24]. Animals were maintained at the University of North Texas Health Science Center (UNTHSC), Fort Worth, TX. All animal experiments were carried out in accordance with and approved by University of North Texas Health Science Center (UNTHSC) Institutional Animal Care and Use Committee (IACUC) approved protocol # 2010/11-14.

### RLIP76 phosphorothioate DNA preparation

The region spanning amino acid residues 171 to 185 (nucleotides 510–555 starting from 1 AUG codon in the open reading frame) in the NH_2_-terminal region of RLIP76 was chosen as the target region for synthesis of phosphorothioate DNA. The oxygen in the backbone of the DNA molecules was replaced by sulfur in each phosphate group, which makes the DNA backbone resistant to nucleases. However, the macromolecule remains electrically charged, impeding its passage across cell membrane. The selected DNA sequence was subjected to BLAST search (National Center for Biotechnology Information database) against expressed sequence tag libraries to ensure that only the selected gene was targeted. Chemically synthesized phosphorothioate DNA in desalted form was purchased from Biosynthesis, Inc., (Lewisville, TX). A 21-nucleotide-long scrambled phosphorothioate DNA was used as a control. The scrambled DNA sequence was not homologous with RLIP76 cDNA in a BLAST search against RLIP76. The targeted cDNA sequence (AAGAAAAAGCCAATTCAGGAGCC) corresponds to nt^508–528^. The corresponding phosphorothioated DNA sequence was GGCTCCTGAATTGGCTTTTTC. The sequence of the scrambled DNA was CATCGAAATCGTTGCAGTTAC. Transfection of phosphorothioate DNA was done using Maxfect transfection reagent (MoleculA) and assayed for silencing 24 hours after transfection. Twelve weeks old C57BL/6 mice born of heterozygous×heterozygous mating were genotyped by PCR [24], [25]. Glucose, cholesterol, and triglycerides measurements in blood serum were performed on RLIP76 wild type (RLIP76^+/+^) and RLIP76 homozygous knock-type (RLIP76^−/−^) animals sacrificed 24 h after a single *i.p*. injection of scrambled or RLIP76-antisense equivalent to 200 µg.

### Effect of RLIP76 antisense, rosiglitazone, metformin, gemfibrozil and atorvastatin

All animal experiments were carried out in accordance with an IACUC approved protocol. For these studies, we have obtained data on the expression of the various signaling proteins in tissues of mice with both genotypes without or with treatment with a single dose of drug shown to cause a significant detectable effect in the therapeutic endpoint in the wild-type animals. Mice were randomly assigned to various groups. Each experimental group had range from 5–7 animals with the number of animals determined by the known accuracy and precision of the assay. For the 5 drugs, 2 genotypes (RLIP76^+/+^ and RLIP76^−/−^ mice), and 2 drug levels (without or with) a minimum of 20 experimental groups were compared for each measured parameter. The number of animals in each experimental group was determined on the basis of a desired ability to distinguish 20% difference in the parameter measured, with a type 1 error of 0.05 and power of 0.90.

The drugs tested in the animals were RLIP76 antisense (6 mg/kg b.w.), rosiglitazone (10 mg/kg b.w.), metformin (250 mg/kg b.w.), gemfibrozil (100 mg/kg b.w.), and atorvastatin (80 mg/kg b.w.). Student's t test was used to compare the effects of the drugs among the groups. ANOVA was performed to check if there is any overall difference among different groups of mice treated with different drugs.

### Western blot analysis

The control and treated mouse liver tissue were homogenized and analyzed by Western-blot analyses for RLIP76, pPARγ, pPARα, pAMPK, JNK, pJNK, pAkt, and HMGCR by using specific antibodies. Briefly, crude fraction containing ∼50 µg of proteins were subjected to sodium dodecyl sulfate–polyacrylamide gel electrophoresis (SDS-PAGE) and proteins were transferred onto nitrocellulose membrane. After blocking with 5% non-fat dry milk, the membrane was incubated overnight with the desired primary antibody (∼1∶1000 dilution). Subsequently, the membrane was incubated with appropriate secondary antibody for 2 h, and the immune-reactive bands were visualized using the enhanced chemiluminescence kit from Perkin-Elmer (Waltham, MA) according to the manufacturer's instructions. The same membrane was reprobed with the antibody against GAPDH (∼1∶5000 dilution) as an internal control for equal protein loading. The intensity of immune-reactive bands on Western blots was determined using a densitometer (Molecular Dynamics, Sunnyvale, CA) equipped with Image QuaNT software.

### Transport of ^3^H-GSHNE (glutathione-conjugate of 4-hydroxynonenal) by RLIP76 and its inhibition by rosiglitazone or metformin

For these experiments, fixed amount of purified rec-RLIP76 (250 ng protein/30 µl reaction mixture) was reconstituted into proteoliposomes, and incubated with rosiglitazone or metformin (concentration ranging from 0–100 µM) for 30 min at 37°C. Effects of rosiglitazone or metformin on RLIP76-mediated ^3^H-GSHNE transports were measured according to a well established and published protocol [24]. In one control rosiglitazone or metformin were excluded while in other control equivalent amount of BSA were reconstituted in proteoliposomes. ATP-dependent uptake of ^3^H-GSHNE (specific activity ∼3.4×10^4^ cpm/nmol, use 10 µM final concentration) were determined by subtracting the radioactivity (cpm) of the control without ATP from that of the experimental containing ATP, and the transport of GS-HNE was calculated in terms of nmol/min/mg protein. Each determination was performed in triplicate.

### Quantification of glucose and lipid levels in RLIP76^+/+^ and RLIP76^−/−^ mice

All animal experiments were carried out in accordance with an IACUC approved protocol. Twelve weeks old C57BL/6 mice born of heterozygous×heterozygous (RLIP76^+/−^×RLIP76^+/−^) mating were genotyped by PCR strategy on mouse tail DNA using forward, reverse and long terminal region (LTR) primers. These mice were commissioned from Lexicon Genetics and were created using Cre-Lox technology [24]. Glucose and lipids measurement in blood serum were performed on wild type (RLIP76^+/+^) and RLIP76 knockout (RLIP76^−/−^) animals after a single oral dose by gavage of rosiglitazone, metformin, gemfibrozil, and atorvastatin equivalent to 10, 250, 100, and 80 mg/kg b.w., respectively.

### Comparison of the therapeutic effects of rosiglitazone, metformin, gemfibrozil and atorvastatin between RLIP76^+/+^ and RLIP76^−/−^ mice

Animals were obtained from our colonies and isolated for 1 week prior to studies in metabolic cages containing 4 animals per cage in a temperature controlled environment with a 12-h light and dark cycle and given water *ad libitum*. We have designed animal studies because mice are an excellent established model for studies of MSy drugs, and there is no alternative means of obtaining the same information. The studies are designed to be directly informative regarding the hypothesis that, lack of RLIP76 will abolish the activity of many drugs used to treat MSy. Animals were weighed and randomized to treatment groups. The drugs were dissolved in appropriate solvents as indicated by MSDS information, diluted further in either corn oil or saline and given by oral gavage in 200 µl final volume. RLIP76^+/+^ and RLIP76^−/−^ animals were treated by oral gavage with a single dose (200 µl) of either diluent alone or diluent containing rosiglitazone (10 mg/kg b.w.), metformin (250 mg/kg b.w.), gemfibrozil (100 mg/kg b.w.) or atorvastatin (80 mg/kg b.w.). Animals were sacrificed with CO_2_ asphyxiation at specified time points after drug dosing, and subjected to complete autopsy with collection of tissues and blood. We used optimal time point to compare the effect of each drug for both RLIP76^+/+^ and RLIP76^−/−^ mice. These studies were designed to measure the hypoglycemic and hypolipidemic effects of these medications.

### Comparison of the expression and signaling functions of PPARγ, AMPK, HMGCR, and PPARα between RLIP76^−/−^ vs. RLIP76^+/+^ mice

For these studies, we compared tissue levels of expression, and signaling functions of PPARγ, HMGCR, AMPK and PPARα between RLIP76^−/−^ and RLIP76^+/+^ mice by Western-blot analyses as well as ABC staining of paraffin embedded liver tissue sections [27].

### Phosphoenolpyruvate carboxykinase (PEP-CK), Fructose 1, 6-bisphosphatase (F-1, 6-BPase) and glucose-6-phosphatase (G6Pase) activity in RLIP76^−/−^ and RLIP76^+/+^ mouse liver

The method of Opie and Newsholme [28] was used for PEP-CK assay. F-1, 6-BPase was assayed by the method of Taketa and Pogell [29]. G6Pase activity was determined using the method of Gierow and Jergil [30].

### Statistical Methods

All data were evaluated with a two-tailed unpaired student's t test or compared by one-way ANOVA and are expressed as the mean ± SD. For *in vivo* studies, drug-treatment values were compared with the vehicle-treatment control values. A *p* value<0.05 was considered statistically significant.

## Results

### The hypoglycemic effect of rosiglitazone is absent in RLIP76^−/−^ mice

**Figure 1:**
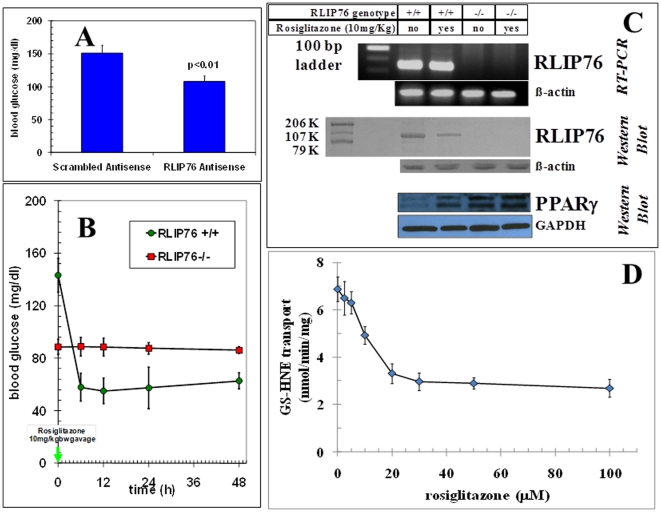
Differential effect of rosiglitazone in RLIP76^**+**/**+**^ *vs.* RLIP76^−/−^ mice. **Panel A:** Effect of RLIP76 depletion by RLIP76 antisense on BG in RLIP76^+/+^ mice. p<0.01, when compared to scrambled antisense treatment. **Panel B:** BG was measured prior to and after a single oral dose of rosiglitazone (10 mg/kg b.w.) by gavage at various time points. p<0.001, when compared between RLIP76^+/+^ and RLIP76^−/−^ mice, and rosiglitazone treatment in RLIP76^+/+^ mice. **Panel C:** Effect of rosiglitazone on RLIP76 expression (by QRT-PCR), RLIP76 protein content, and PPARγ protein content (by Western blot) in mouse liver tissue lysates. GAPDH expression was used as loading control. **Panel D:** Inhibition of the transport activity of purified recombinant human RLIP76 towards physiological substrate ^3^H-GSHNE by rosiglitazone. In panels A & B, 5 mice per group were used. These experiments were repeated three times and similar results were obtained.

We performed studies comparing effects of rosiglitazone on blood glucose (BG). Consistent with previous studies, RLIP76^−/−^ mice had significantly lower baseline BG (87±8 mg/dl) as compared with the RLIP76^+/+^ mice (158±10 mg/dl) (p<0.01) [1]. Also confirming previous studies, administration of a single 200 µg dose of RLIP76-antisense (R508) i.p. that has been shown to cause >90% depletion of hepatic RLIP76 within 24 h [31]–[34], caused a drop in BG in RLIP76^+/+^ mice (**Fig. 1A**). Remarkably, no effect was seen in RLIP76^−/−^ mice. Rather than an accentuated hypoglycemic affect as would have been predicted if rosiglitazone operated through a mechanism independent of RLIP76, we observed a striking and complete lack of action of rosiglitazone in RLIP76^−/−^ mice (**Fig. 1B**). In response to rosiglitazone, hepatic mRNA expression of Ralbp1 (mouse gene encoding the splice variant protein, RLIP76) was unaffected, but cellular RLIP76 protein was depleted to less than 50% within 24 h of rosiglitazone. Rosiglitazone caused an expected induction of PPARγ in RLIP76^+/+^ mice. In stark contrast, PPARγ protein levels were increased in RLIP76^−/−^ mouse liver even in the absence of rosiglitazone and were not affected further by rosiglitazone (**Fig. 1C**). The possibility that rosiglitazone was a direct inhibitor of the GS-HNE transport activity of RLIP76 was confirmed using purified and authenticated recombinant human RLIP76 reconstituted in artificial proteo-liposomes to measure transport activity towards ^3^H-GSHNE at varying concentrations of rosiglitazone (**Fig. 1D**). These findings strongly support a model in which rosiglitazone's hypoglycemic actions are mediated by inhibition of RLIP76, and a consequent activation of PPARγ as a result of increased oxidative stress due to depleted RLIP76 activity.

### The hypoglycemic effect of metformin is absent in RLIP76^−/−^ mice

**Figure 2:**
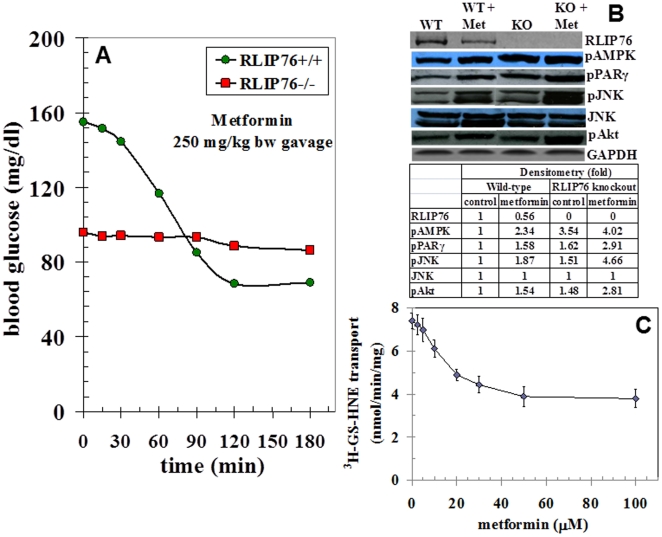
Differential effect of metformin in RLIP76^**+**/**+**^ and RLIP76^−/−^ mice. **Panel A:** BG was measured prior to and after a single oral dose of metformin (250 mg/kg b.w.) by gavage at various time points (n = 6 mice/group). p<0.001, when compared between RLIP76^+/+^ and RLIP76^−/−^ mice, and metformin treatment in RLIP76^+/+^ mice. **Panel B:** Effect of metformin on RLIP76, pAkt, pJNK, PPARγ, and pAMPK expression by Western blot in RLIP76^+/+^ and RLIP76^−/−^ control and metformin treated mouse liver tissue lysates, and developed bands were quantified by scanning densitometry. GAPDH expression was used as loading control. **Panel C:** Inhibition of the transport activity of purified rec-RLIP76 towards ^3^H-GSHNE by metformin. The experiment was repeated twice and similar results were obtained. WT, wild-type (RLIP76^+/+^); KO, RLIP76-knockout (RLIP76^−/−^); Met, metformin.

**Figure 3:**
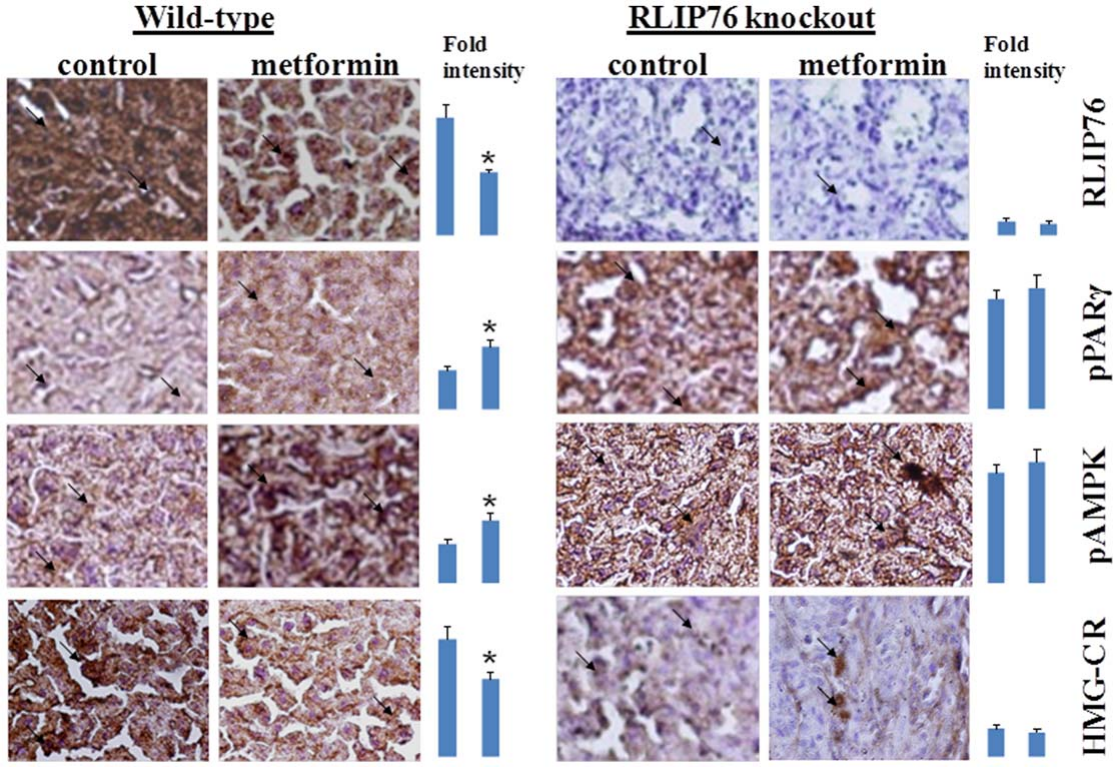
Effect of metformin on RLIP76, PPARγ, AMPK, and HMGCR expression in paraffin embedded RLIP76^**+**/**+**^ and RLIP76^−/−^ mouse liver tissues section by immuno-histochemistry using ABC staining kit (Vector). Immuno-reactivity is evident as a dark brown stain, whereas non-reactive areas display only the background color. Sections were counter-stained with Hematoxylin (blue). Photographs at 40× magnification were acquired using Olympus Provis AX70 microscope. Percent staining was determined by measuring positive immuno-reactivity per unit area. Arrows represent the area for positive staining for an antigen. The intensity of antigen staining was quantified by digital image analysis. Bars represent mean ± S.E. (n = 5 sections from different animals); * p<0.01 compared with control.

The general applicability of our hypothesis that peripherally acting hypoglycemic drug will fail in RLIP76^−/−^ mice is based on observed lack of clathrin-dependent endocytosis (CDE) in the absence of RLIP76, and consequent global disruption of receptor kinases and other signaling down-stream of receptor-ligand pairs [1], [25], [35]. Prolonged signaling by insulin bound to the plasma membrane receptor due to the lack of CDE, and consequent insulin-sensitivity has been shown to play a key role in the baseline hypoglycemia, but the most relevant kinase pathways involved were not elucidated. AMPK is an important kinase for the control of metabolism of glucose and lipids, and is known to regulate CDE [36], [37]. Because the biguanide class oral hypoglycemic, metformin, acts through AMPK as well as PPARγ, we conjectured that it would also fail to function in RLIP76^−/−^ mice. Rather than an accentuated hypoglycemic affect as would have been predicted if metformin operated through a mechanism independent of RLIP76, we observed a striking and complete lack of action of metformin in RLIP76^−/−^ animals (**Fig. 2A**). RLIP76 protein was depleted significantly to less than 50% within 2 h of metformin, and as expected, there was induction of pAkt, pJNK, pAMPK and PPARγ in RLIP76^+/+^ mice. In contrast, the levels of pAkt, pJNK, pAMPK and PPARγ were increased at baseline in RLIP76^−/−^ mice liver, even in the absence of metformin, and were affected further by metformin (**Fig. 2B**). Transport activity of purified recombinant human RLIP76 towards the model physiological substrate, ^3^H-GSHNE, was inhibited in a dose-dependent manner, with a saturable behavior, indicating that metformin inhibits only half of the transport activity (**Fig. 2C**) which was an observation consistent with that found with physiological inhibitors of RLIP76 transport activity, POB1, Hsf-1, and cdc-2 (CDK-1) [38], [39]. These results were further confirmed by immuno-histochemical studies showing that metformin caused expected increase in PPARγ and pAMPK, and a decrease in HMG-CoA reductase (HMGCR) as well as RLIP76. RLIP76 was absent, and PPARγ and pAMPK were maximally induced in RLIP76^−/−^ mice, and no further effect of metformin was found. Most remarkably, HMGCR was markedly deficient in RLIP76^−/−^, explaining the observed hypocholesterolemia in these animals (**Fig. 3**). Taken together, these findings strongly support that RLIP76 is necessary for the hypoglycemic effect of metformin, and indicate that direct inhibition of RLIP76 by metformin is the mechanism. RLIP76^−/−^ mice have baseline hypoglycemia, and baseline increases in pAMPK, PPARγ, pJNK, and pAKT, which provides perhaps the reason for their hypoglycemia. If this were true, further activation of these signals by metformin, (as is seen in RLIP76^−/−^ mice), should cause further hypoglycemia – but it clearly does not. These findings are perhaps best understood in the context of previous studies showing that RLIP76^−/−^ mice have markedly elevated levels of lipid-peroxidation and consequent elevation of lipid hydroperoxides and their degradation products [23]–[25]. Although such alterations are strongly clinically linked to insulin-resistance and metabolic syndrome, the precise opposite findings are seen in the RLIP76^−/−^ mice: insulin-sensitivity, hypoglycemia, hypolipidemia and fat-loss. That is, oxidative stress is insufficient to cause insulin-resistance in the absence of RLIP76. Because of the recently established role of RLIP76 as a mediator of CDE through its activity as an ATP-dependent efflux transporter of glutathionylated derivatives of lipid-peroxidation, the established role of CDE in mediating insulin-resistance [23], [24], [40]–[42] and in the context of present findings, we conclude that insulin-resistance should result from an abundance of RLIP76, as occurs under stress-conditions. Thus, under stress conditions, increased expression and activity of RLIP76 causes increased CDE, which antagonizes insulin-induced hypoglycemic effects leading to insulin-resistance. In the absence of RLIP76, the CDE-mediated antagonism is absent and insulin-sensitivity results, presumably as a result of failure to suppress PPARγ or AMPK. According to this postulate, drugs which inhibit the transport activity of RLIP76 should reduce CDE allowing more prolonged insulin action at the plasma membrane. These findings strongly support a model in which metformin's hypoglycemic actions are mediated by inhibition of RLIP76, and a consequent activation of AMPK and PPARγ as a result of increased oxidative stress due to depleted RLIP76 activity. PPARγ and RLIP76 appear to be mutually inversely regulated, and baseline activation of AMPK and PPARγ are the specific down-stream kinases whose activation is increased with the loss of CDE.

### Down-regulation of gluconeogenic enzymes in RLIP76^−/−^ mice

**Figure 4:**
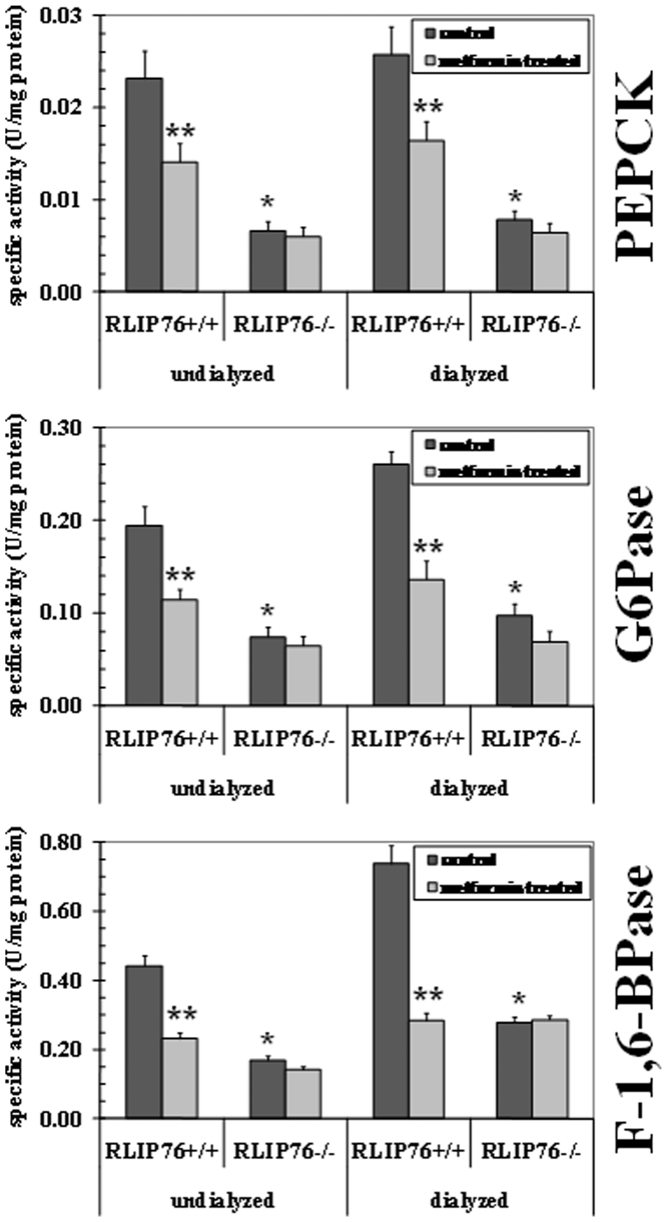
The activity of gluconeogenesis enzymes. The activity of PEPCK, F-1, 6-BPase, and G6Pase was tested in un-dialyzed and dialyzed liver homogenates of control and metformin treated RLIP76^+/+^ and RLIP76^−/−^ mice (n = 3) as protocols standardized by us [1]. *p<0.001, when compared to RLIP76^+/+^, and **p<0.005, when compared with metformin treatment in RLIP76^+/+^. The enzyme PEPCK, catalyze the conversion of phosphoenolpyruvate to fructose 1,6-biphosphate in a series of steps involving oxidation of NADH to NAD. In this assay, the loss of NADH was determined spectrophotometrically by measuring absorbance at 340 nm, based on the method of Opie and Newsholme [28]. To detect F-1, 6-BPase activity, a spectrophotometric coupled enzyme assay was used by a method of Taketa and Pogell [29]. F-1, 6-BPase activity was coupled with phosphoglucose isomerase and NADP dependent glucose 6-phosphate dehydrogenase, and NADPH formation was measured at 340 nm. G6Pase activity was determined spectrophotometrically using the method of Gierow and Jergil [30]. The method is based on a coupled enzyme reaction in which glucose formed is reacted with glucose oxidase and peroxidase and the quinoneimine formed is a colored product and its formation can be followed spectrophotometrically at 510 nm.

The hypoglycemic effects of metformin or rosiglitazone occurs through the activation of AMPK and/or PPARγ, which results in the inhibition of hepatic glucose production by down-regulation of gluconeogenic targets (PEPCK, F-1,6-Bpase, and G6Pase). The activity of key gluconeogenic enzymes, glucose-6-phosphatase (G6Pase), fructose-1,6-bisphosphatase (F1,6-BPase) and phosphoenolpyruvate carboxy-kinase (PEPCK) were already significantly decreased in RLIP76^−/−^ mouse liver even in the absence of any hypoglycemic drug [1]. Indeed, treatment of RLIP76^+/+^ mice with metformin also decreased these enzymes in the liver (**Fig. 4**). Comparison of these enzyme activities in RLIP76^+/+^ *vs.* RLIP76^−/−^ mouse liver homogenate without or with overnight dialysis showed that dialyzable inhibitors were present, but could not alone account for the lower activity of these enzymes in RLIP76^−/−^. 4-HNE, an alkenal shown to increase ∼3 fold in RLIP76^−/−^ mouse liver, did not directly affect the activity of PEP-CK or G6Pase, but activated F-1,6-BPase activity.

### Hypocholesterolemic effect of atorvastatin is absent in RLIP76^−/−^ mice

Atorvastatin induces hypocholesterolemia through inhibition of HMG-CoA reductase [43]. Products of HMG-CoA reductase are critical not only for cholesterol biosynthesis, but also for the synthesis of farnesyl and geranyl groups that are necessary for directing proteins including Ras, Ral and Rho to plasma membrane. RLIP76 was originally shown to be a functional link between the Ras and Ral pathway and is directly regulated by Ral in the mechanism of action of heat-shock protein, and regulates Rho through its GAP activity [44], [45]. Thus, it is possible that one of the consequences of HMG-CoA reductase inhibition could be the impaired function of RLIP76. However, because RLIP76^−/−^ animals are relatively hypocholesterolemic, it is possible that HMG-CoA reductase is inhibited or depleted as a direct or indirect consequence of the loss of RLIP76 (perhaps by lipid-peroxidation products).

**Figure 5:**
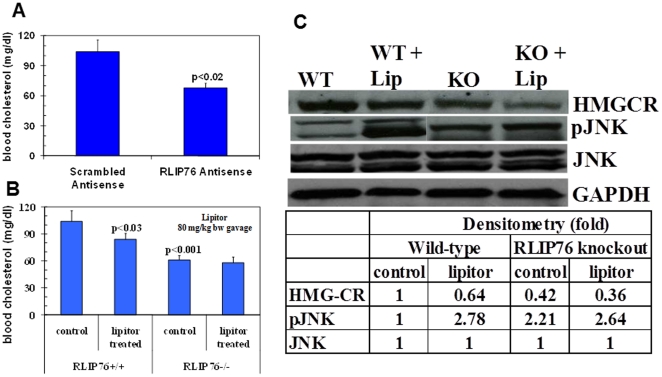
Differential effect of atorvastatin (lipitor) in RLIP76^**+**/**+**^ and RLIP76^−/−^ mice. **Panel A:** Effect of RLIP76 depletion by RLIP76 antisense on cholesterol level in RLIP76^+/+^ mice. p<0.02, when compared to scrambled antisense treatment. **Panel B:** cholesterol level was measured prior to and 24 h after a single oral dose of atorvastatin (80 mg/kg b.w.) by gavage in RLIP76^+/+^ and RLIP76^−/−^ mice. p<0.001, when compared between RLIP76^+/+^ and RLIP76^−/−^ mice, and p<0.03 when compared to lipitor treatment in RLIP76^+/+^ mice. In panels A & B, 6 mice per group were used. **Panel C:** Effect of atorvastatin on pJNK and HMGCR expression by Western blot in mouse liver tissue lysates, and developed bands were quantified by scanning densitometry. GAPDH expression was used as loading control. WT, wild-type; KO, RLIP76-knockout; Lip, Lipitor.

The most frequently prescribed hypocholesterolemic agent, atorvastatin (Lipitor) functions through specific HMGCR inhibition [43]. HMGCR catalyzes the rate-determining step of cholesterol biosynthesis and it has been demonstrated that inhibitors of HMGCR effectively lower plasma cholesterol levels. The loss of HMGCR in RLIP76^−/−^ mice is also of key significance and suggested that HMGCR inhibition should cause no further lowering of cholesterol in RLIP76^−/−^ mice. This was indeed shown to be the case. Administration of RLIP76 antisense in the RLIP76^+/+^ mouse (at a dose known to lower tissue levels of RLIP76 protein to <10%) caused a significant drop in blood cholesterol from 104 to 68 mg/dl (**Fig. 5A**).

Cholesterol levels were significantly lower in RLIP76^−/−^ as compared with RLIP76^+/+^ mice. Atorvastatin treatment caused no detectable hypocholesterolemic effect in the RLIP76^−/−^ mouse (**Fig. 5B**). Atorvastatin treatment reduced hepatic HMGCR in both RLIP76^+/+^ and RLIP76^−/−^ mice, but activation of JNK was lacking in RLIP76^−/−^ mice (**Fig. 5C**). Because HMGCR inhibitors directly control the rate of cholesterol synthesis, it should further lower cholesterol levels in RLIP76^−/−^ mice, but this is clearly not the case (**Fig. 5A and B**). The alternative explanation seems more valid that RLIP76 is an effector functionally down-stream of HMGCR (because RLIP76 is regulated by Ral, which determines its sub-cellular localization) which is necessary for the hypocholesterolemic effect of atorvastatin. RLIP76 can be considered functionally down-stream of HMG-CoA because of Ral, which regulates RLIP76 sub-cellular localization, and Rho, which is inhibited by RLIP76. Indeed, cdc42, towards which RLIP76 displays GAP activity, is among the proteins whose activities are impaired by HMGCR inhibitors [44].

### Hypotriglyceridemic effect of gemfibrozil due to constitutive activation of PPARα

**Figure 6:**
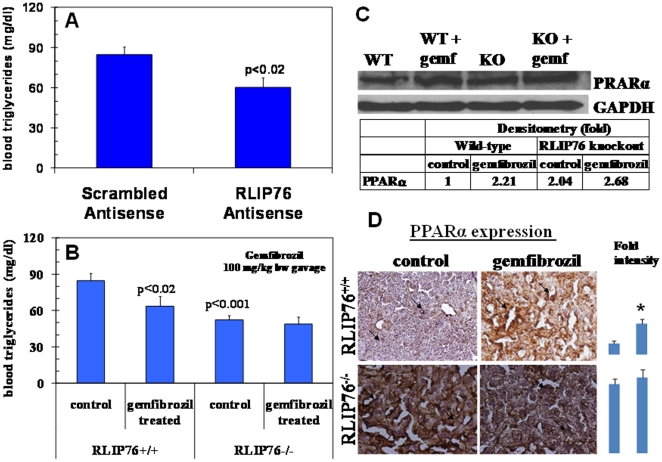
Differential effect of gemfibrozil in RLIP76^**+**/**+**^ and RLIP76^−/−^ mice. **Panel A:** Effect of RLIP76 depletion by RLIP76 antisense on triglycerides level in RLIP76^+/+^ mice. p<0.02, when compared to scrambled antisense treatment. **Panel B:** triglycerides level was measured prior to and 24 h after a single oral dose of gemfibrozil (100 mg/kg b.w.) by gavage in RLIP76^+/+^ and RLIP76^−/−^ mice. p<0.001, when compared between RLIP76^+/+^ and RLIP76^−/−^ mice, and p<0.02 when compared to gemfibrozil treatment in RLIP76^+/+^ mice. In panels A & B, 5 mice per group were used. **Panel C:** Effect of gemfibrozil on PPARα expression by Western blot in mouse liver tissue lysates, and developed bands were quantified by scanning densitometry. GAPDH expression was used as loading control. WT, wild-type; KO, RLIP76-knockout; Gemf, gemfibrozil; **Panel D:** Effect of gemfibrozil on PPARα expression in paraffin embedded RLIP76^+/+^ and RLIP76^−/−^ mouse liver tissues section by immuno-histochemistry using ABC staining kit (Vector). Immuno-reactivity is evident as a dark brown stain, whereas non-reactive areas display only the background color. Sections were counter-stained with Hematoxylin (blue). Photographs at 40× magnification were acquired using Olympus Provis AX70 microscope. Percent staining was determined by measuring positive immuno-reactivity per unit area. Arrows represent the area for positive staining for an antigen. The intensity of antigen staining was quantified by digital image analysis. Bars represent mean ± S.E. (n = 5); * p<0.002 compared with control.

Administration of a single 200 µg dose of RLIP76-antisense, i.p., that has been shown to cause >90% depletion of hepatic RLIP76 within 24 h [31]–[34], caused a drop in triglycerides in RLIP76^+/+^ mice (**Fig. 6A**). Remarkably, no effect was seen in RLIP76^−/−^ mice. Gemfibrozil is an oral drug used to lower lipid levels in the blood. Gemfibrozil increases the activity of PPARα, a receptor that is involved in the metabolism of carbohydrates and fats, and reduce triglyceride level. If gemfibrozil operated through a mechanism independent of RLIP76, an accentuated hypotriglyceridemic effect would have been observed in RLIP76^−/−^ mice. In contrast, we observed a striking and complete lack of action of gemfibrozil in RLIP76^−/−^ mice (**Fig. 6B**). PPARα is inducible by the lipid-hydroperoxidation product of ω-6 fatty acid, in particular leukotrienes B_4_ (LTB_4_), the direct precursor of its glutathionylated metabolite, LTC_4_ (which is metabolized to mercapturic acid and is a known substrate for efflux by RLIP76) [46] (**Fig. 6C**). Thus, a potential direct mechanism exists that could explain hypotriglyceridemia. We found that PPARα was indeed constitutively activated in the tissues of the RLIP76^−/−^ mice (**Fig. 6D**). In such a case, gemfibrozil should have no further triglyceride depleting effects. However, the effects of gemfibrozil have also been directly linked to inhibition of fatty acid synthesis enzymes, suggesting that their effects may not be completely abrogated. The differential effects of these drugs on lipid particle distribution (increasing HDL and lowering VLDL) appears more likely due to direct or indirect effects on CDE, which plays a critical role in lipid trafficking. So we conclude that hypotriglyceridemia in RLIP76^−/−^ mice are due largely or entirely to PPARα accumulation.

Just as PPARγ is constitutively increased in RLIP76^−/−^ mice and may contribute solely or partially to hypoglycemia, it is possible that the observed hypo-triglyceridemia seen in RLIP76^−/−^ mice is due to constitutive activation of PPARγ. Mechanistic rationale for this possibility could be based on the fact that leukotrienes B_4_, a physiological ligand for activation of PPARα, is also a mercapturic acid precursor (through leukotrienes C_4_), and would be expected to be increased in RLIP76^−/−^ mice. Because hypotriglyceridemic agents such as gemfibrozil function largely through activation of PPARα, their actions could also be deficient in RLIP76^−/−^.

## Discussion

The striking absence of hypoglycemic function of rosiglitazone and metformin, and hypolipidemic function of gemfibrozil and atorvastatin (Lipitor) in RLIP76^−/−^ mice has broad and significant impact on our understanding of the mechanisms involved in mediating and treating metabolic syndrome. RLIP76, a glutathione-conjugate transporter, may play a central role in oxidative-stress-associated hyperglycemia and hyperlipidemia. RLIP76^−/−^ mice are extremely insulin sensitive, and have baseline BG, cholesterol and triglyceride levels that are about one-half of that in wild-type mice [1]. Depletion of RLIP76 by antisense or inhibition by antibodies decreases blood glucose, triglycerides and cholesterol. In contrast, the insulin-sensitizing thiazolidinedione drug, rosiglitazone, and metformin (Glucophage) is a widely used drug for T2D treatment, had absolutely no effect on the blood glucose of RLIP76^−/−^ mice. Present studies indicate that this lack of action is because PPARγ and AMPK are already maximally induced in RLIP76^−/−^ mice and that PPARγ and AMPK activation in RLIP76^+/+^ is associated with inhibition of the transport activity of RLIP76.

Present studies also indicate that in RLIP76^+/+^ mice, rosiglitazone inhibits the transport activity of RLIP76 and causes depletion of RLIP76 protein without affecting the level of mRNA, and induces PPARγ. In stark contrast, PPARγ is maximally activated in RLIP76^−/−^ mice even in the absence of rosiglitazone, treatment with which causes absolutely no further effect on PPARγ level. That is, PPARγ activation correlates with RLIP76-inhibition, and RLIP76-inhibition leads invariably to inhibition of CDE; thus, PPARγ activation should vary inversely with CDE. The link between endocytosis and PPARγ is also apparent from the known mechanism of action of another hypoglycemic agent, metformin, which functions through inhibition of AMPK, a kinase that regulates the rate of endocytosis, and also affects PPARγ through PGC1α [36], [37], [47]. Because CDE plays a crucial role in cholesterol trafficking, and because the Ral-pathway is known to be critically regulated by products synthesized through HMG-CoA [43], it is possible that the hypocholesterolemic effect of HMGCR inhibition will be diminished in the RLIP76^−/−^ mice. CDE also plays a crucial role in trafficking of triglycerides, and because hypotriglyceridemic agents such as gemfibrozil operate through PPARα [48], it is possible that these agents will also fail to exert their therapeutic effect.

Our studies have characterized that RLIP76 represents a critical and determinant node of signaling that regulates the MSy incidence and response to interventional strategies. The JNK1 activation is a known factor that regulates the insulin sensitivity [49]. Some studies have reported that JNK1 knock out mice have marked resistance to high fat induced obesity [50]. Further studies in JNK1 knock-out mice would unveil the precise cross-talk between RLIP76 and JNK1 and whether RLIP76 can also act independent of JNK1 in regulating the incidence of MSy. Besides its potential role in the incidence of MSy, RLIP76 has emerged as a mechanistically significant and effective interventional target for MSy. According to a prospective study, metformin used for the management of MSy also reduced the incidence of cancer-risk [51], [52]. RLIP76 is an established factor essential for the incidence and progression of multiple cancers and RLIP76 knock-out mice are resistant to chemical neoplasia [31]–[35]. Thus, inhibition of RLIP76 by metformin has revealed an RLIP76 dependent mechanism for both the anti-MSy and anti-cancer effects of metformin.

Our recently published studies demonstrate that RLIP76^−/−^ mice used for these studies were found to have marked insulin-sensitivity, and blood glucose was 46% lower than in RLIP76^+/+^ animals (p<0.001). RLIP76^−/−^ mice also had lower total serum cholesterol and triglycerides (43% and 40% of control, respectively; p<0.01) [1]. The hypoglycemia in RLIP76^−/−^ mice is particularly remarkable because markers of oxidative-stress are remarkably increased in the tissues of the RLIP76^−/−^ animals [1], [23]–[25]. Thus, in the absence of RLIP76, increases in these lipid-peroxidation products are insufficient by themselves to turn on any signaling pathway that can increase BG or lipids. Increased gluconeogenesis was particularly remarkable given that the activity of key gluconeogenic enzymes, G6Pase, F1,6-BPase, and PEPCK, in liver of RLIP76^−/−^ mice was significantly inhibited.

Enhanced basal pAMPK levels in RLIP76^−/−^ mice was another salient finding which strengthens the postulate that RLIP76 is a highly effective target for developing interventional strategies for MSy. Resveratrol, commonly used anti-oxidant, is known to activate AMPK which could contribute to its protective effects from high fat diet induced insulin-resistance [53], [54]. AMPK protects cells from stresses that cause ATP depletion by switching off ATP-consuming biosynthetic pathways. AMPK is activated by phosphorylation by an upstream protein kinase known as AMPK kinase. Activated AMPK can phosphorylate and regulate *in vivo* HMG-CoA, which is key regulatory enzyme of sterol synthesis [43], [47]. HMG-CoA limits the rate of cholesterol synthesis in liver tissue. Lipitor, inhibitor of HMG-CoA, exerts anti-inflammatory effects by lowering plasma cholesterol. Activation of AMPK leads to the inhibition of cholesterol synthesis by the phosphorylation of HMG-CoA reductase [43]. Loss of RLIP76 significantly affects the activation of stress and apoptosis pathway proteins [1], [25], [35]. Activation of AMPK leads to the inhibition of cholesterol synthesis by the phosphorylation of HMGCR. AMPK activation would be a good approach to treat T2D. These medications generally function to increase the effectiveness of insulin-mediated postprandial inhibition of hepatic gluconeogenesis. These findings provide a new insight on the mechanisms of action of hypoglycemic and/or hypolipidemic drugs.

RLIP76 knock-out mice survive well and are active. In our extensive and previously published studies, RLIP76 inhibition specifically leads to targeting signaling of importance in diabetes mellitus and other oxidative stress related conditions like cancers where targeting RLIP76 leads to selective cancer cell death without affecting the survival of normal cells and tissues [1], [31]–[33]. Hence, both global and selectively targeted approaches can be reasonably pursued as required while targeting RLIP76. In conclusion, our results suggest that RLIP76 is a key effector controlled by multiple proteins known to regulate the metabolic abnormalities of diabetes and metabolic syndrome, and that in its absence drugs that target these proteins will fail to function. The specific events that regulate the transport-effector/clathrin-endocytosis activity of RLIP76 (i.e. phosphorylation of RLIP76 by JNK, Akt, AMPK) will be explored in the future studies.
